# Bioaccessibility of Phenolic Compounds and Polyunsaturated Fatty Acids in Whole and Ground Chia Seeds and Sprouts

**DOI:** 10.1007/s11130-026-01577-y

**Published:** 2026-10-14

**Authors:** Flávia Oliveira Brito, Lilia Zago, Juliana Delvizio, Carolina Beres, Kim Ohanna Pimenta Inada, Vanessa Naciuk Castelo-Branco, Roberta Fontanive Miyahira

**Affiliations:** 1 Graduate Program in Food, Nutrition, and Health, Institute of Nutrition, State University of Rio de Janeiro, 524, 12◦ andar, sala 12006 D – Maracanã, Rio de Janeiro, RJ CEP: 20550-013 Brazil; 2 Food Biotechnology Laboratory, School of Pharmacy, Fluminense Federal University, Niteroi, Brazil

**Keywords:** Chia, Germination, Gastrointestinal digestion, Phenolic compounds, Rosmarinic acid, Omega-3

## Abstract

This study investigated the effect of grinding and germination on phenolic compounds, fatty acids, and antioxidant capacity of chia seeds after in vitro gastrointestinal digestion. After digestion, the samples ground chia (GC) and ground chia sprouts (GCS) showed the highest values of total phenolic compounds, antioxidant capacity, and fatty acids when compared to the respective whole samples, whole chia (WC), and whole chia sprout (WCS). The total phenolic compounds reached 7.00 mg GAE/g (GC) and 8.76 mg GAE/g (GCS), compared to 4.40 mg GAE/g (WC) and 2.57 mg GAE/g (WCS). In the evaluation of antioxidant capacity, GC and GCS showed higher values in the FRAP (40.38 and 38.62 µmol FS/g), ABTS (79.98 and 74.99 µmol TE/g), and DPPH assays (63.36 and 51.51% AA), compared to WC and WCS (FRAP: 2.85 and 11.53 µmol FS/g; ABTS: 57.40 and 47.98 µmol TE/g; DPPH: 39.27 and 42.11% AA, respectively). In addition, rosmarinic acid was detected only in the ground samples (seeds or sprouts), while rosmarinic acid 3-O-glycoside and caffeic acid were detected only in the ground seeds after digestion. Regarding the lipid content, values of 6.33 g/100 g for GC and 5.36 g/100 g for GCS were obtained, while WC and WCS showed only 0.19 g/100 g. On the other hand, omega-3 was identified in all samples, although its amount decreased in the sprouts. It is concluded that under the evaluated conditions, grinding was a more determining factor than germination in improving the bioaccessibility of phenolic compounds and fatty acids in chia seeds.

**Supplementary Information:**

The online version contains supplementary material available at https://doi.org/10.1007/s11130-026-01577-y.

## Introduction

Chia seeds, obtained from the plant *Salvia hispanica* L., have gained popularity due to their nutritional profile. These seeds have high antioxidant capacity and elevated levels of phenolic compounds, proteins, dietary fibers, and polyunsaturated fatty acids, especially the α-linolenic acid from the omega-3 family [1, 2]. Chia seeds also has antioxidant, anti-inflammatory, antidiabetic, antihypertensive, and anti-obesity properties [3]. Regular consumption of chia seeds has been associated with health benefits, such as improvements in blood pressure and glycemic control [4].

Despite their nutritional composition, consuming whole chia seeds does not mean that their nutrients and bioactive compounds will be released in the gastrointestinal tract. The seed coat represents an important barrier to digestion [5]. Thus, when consumed in their whole form, chia seeds mostly pass through the gastrointestinal tract intact, preventing digestive enzymes from accessing the interior of the seed, where most of the nutrients are concentrated [5].

Processing techniques, such as grinding and germination, can modify the structure of the seeds and consequently influence the bioaccessibility of the nutrients [6, 7]. Grinding reduces the particle size and increases the surface area, facilitating the release of nutrients and phytochemicals from the seed matrix [8]. It has been demonstrated that grinding chia can increase the release of phytochemicals and improve the antioxidant properties of chia flour [7]. During germination, the activation of hydrolytic enzymes occurs, which break down reserve macromolecules and modify the composition of the cellular matrix. As a result, the nutrients can be released from their storage structures and become more accessible to digestive enzymes [9].

Given that the physical structure of the seeds hinders the release of nutrients and phytochemicals, determining whether the combination of germination and grinding can result in better bioaccessibility of these compounds is important, as no information was found in the literature on the behavior of bioactive compounds in ground chia sprouts after simulated gastrointestinal digestion. Therefore, this study aimed to evaluate the effects of grinding and germination on the phenolic compounds, fatty acids, and antioxidant capacity of chia seeds subjected to in vitro gastrointestinal digestion.

## Materials and Methods

The Materials and Methods section is presented as supplementary material.

## Results and Discussion

### Phenolic Compounds and Antioxidant Capacity

Phenolic compounds are synthesized by plants that, although not essential for meeting human nutritional needs, have potential health benefits. This is due to their antioxidant, anti-inflammatory, and cardioprotective properties [10]. Table 1 shows the results for total phenolic compound content and antioxidant capacity in undigested and digested chia seeds and sprouts. Undigested chia seeds had a total phenolic content (TPC) of 1.91 mg/GAE/g/dw, a value similar to those found in previous studies, which ranged from 1.41 to 2.58 mg/GAE/g/dw [2, 11].

**Table 1:** Total phenolic content and antioxidant capacity in undigested and digested chia seeds and sprouts (dry weight - dw)

Method and measures	Undigested		Digested			
	GC	GCS	WC	GC	WCS	GCS
Total phenolics (mg GAE/g)	1.91 ± 0.15^Aa^	1.49 ± 0.03^aC^	4.40 ± 0.20^eB^	7.00 ± 0.03^dB^	2.57 ± 0.05^fD^	8.76 ± 0.31^cD^
FRAP (µmol FS/g)	5.04 ± 0.10^aA^	3.16 ± 0.08^bC^	12.85 ± 1.14^dB^	40.38 ± 1.41^cB^	11.53 ± 0.98^dD^	38.62 ± 1.64^cD^
ABTS (µmol TE/g)	45.65 ± 0.29^aA^	43.18 ± 0.59^bC^	57.40 ± 1.05^eB^	79.98 ± 0.67^cB^	47.98 ± 0.67^fD^	74.99 ± 0.44^dD^
DPPH (%AA)	85.21 ± 1.11^aA^	80.90 ± 1.28^bC^	39.27 ± 1.39^eB^	63.36 ± 0.45^cB^	42.11 ± 1.79^eD^	51.51 ± 0.88^dD^

*WC* whole chia, *WCS* whole chia sprout, *GC* ground chia, *GCS* ground chia sprout, *FRAP* Ferric reducing antioxidant power, *ABTS* Trolox-equivalent antioxidant capacity, *DPPH* 1,1-diphenyl-2-picrylhydrazyl radical. Values are expressed as mean ± SD. Undigested samples: different lowercase letters (a, b) in the same row indicate statistically significant differences (*p* < 0.05), according to the unpaired Student’s *t*-test. Digested samples: Different lowercase letters (c, d, e, f) in the same row indicate statistically significant differences (*p* < 0.05) according to one-way nonparametric ANOVA and Tukey’s post hoc test. Uppercase letters on the same row indicate a statistically significant difference (*p* < 0.05) between the digested sample and the corresponding undigested sample, according to the unpaired Student’s t-test

Although some studies have shown that chia seed germination was able to increase the TPC [11, 12], this increase was not observed in the present study (Table 1). Similarly, Menezes et al. [13], who also used Brazilian chia seeds and a germination protocol similar to the present study, did not observe an increase in TPC after chia germination. A divergent result was also found in the study by Salgado et al [2], in which germination led to a significant decrease in TPC. However, these differences between studies may be associated with various factors, such as the initial characteristics of the seed and the experimental conditions used in the study. In addition, germination time and environmental factors—including temperature, salinity, light, and humidity—can directly affect sprout metabolism and the synthesis of bioactive compounds [14]. The digestive process can promote the release of phenolic compounds from the food matrix, resulting in increased bioaccessibility of these compounds [15]. This behavior was observed in the present study, as all digested samples had higher TPC content than the undigested samples. A similar result was found by Calvo-Lerma et al [6], who demonstrated that the bioaccessible TPC values in chia seeds and sprouts are higher than those in undigested samples.

However, the grinding process appears to be a more decisive factor in the bioaccessibility of these molecules. After gastrointestinal digestion, the ground samples (seed and sprout) exhibited higher TPC values (7.00 and 8.76 mg/GAE/g/dw, respectively) than the whole seed and sprout samples (4.40 and 2.57 mg/GAE/g/dw, respectively), indicating that grinding facilitated the release of these compounds. Similarly, Calvo-Lerma et al [6] found that digested whole seeds contained 1.81 mg/GAE/g of TPC, while digested ground chia reached 6.13 mg/GAE/g. A similar pattern was observed by Labanca et al [7]. The authors found TPC values of 0.42 mg/GAE/g for the digested whole seeds and 4.68 mg/GAE/g for the digested ground seeds. These results demonstrate that grinding chia seeds promoted a greater release of total polyphenols following the digestive process, since reducing the particle size of the seeds leads to an increase in the surface area exposed to digestive enzymes and gastrointestinal fluids. Consequently, there is a greater release of phenolic compounds that were previously retained in the food matrix [16]. It is important to note that no studies that simulated the gastrointestinal digestion of ground chia sprouts were found.

Table 1 shows the results of the antioxidant capacity of the samples, as assessed by three different methods: ABTS, DPPH, and FRAP. Undigested chia seeds exhibited higher antioxidant capacity in all tested methods compared to undigested sprouts. This result differs from other studies that observed a significant increase in antioxidant capacity resulting from the germination of chia seeds, regardless of the method used [2, 12].

The antioxidant capacity of chia after digestion varied depending on the analytical method used. While the ABTS and FRAP assays showed an increase in the antioxidant capacity of the samples (seeds and sprouts), the DPPH assay showed a decrease after the gastrointestinal digestion process, which may indicate that the compounds released during digestion have a lower affinity for the specific radicals in this method. This characteristic post-digestive behavior has been observed in other studies on chia. Hernández-Olivas et al [17] found a significant increase in ABTS values but observed a decrease in the DPPH and FRAP assays following the digestion of chia samples. Meanwhile, Pellegrini et al. [16] found a significant increase (*p* < 0.05) in antioxidant activity using the ABTS, FRAP, and DPPH methods on the digested chia samples. As observed for TPC, mechanical breakdown by grinding appears to be an important factor in increasing the antioxidant capacity of the samples. After digestion, the ground seeds exhibited the highest FRAP, ABTS, and DPPH values, followed by the ground sprouts (Table 1). Despite the significant differences in phenolic compounds after digestion in the crushed samples (WC and WCS), the results of the antioxidant capacity obtained by the FRAP and DPPH methods were similar. These results may possibly be related to other metabolites generated during the digestive process, in addition to other compounds, such as aromatic amino acids [18]. FRAP, DPPH, and ABTS assays are widely used in studies of bioactive compounds because of their practicality, low cost, and operational simplicity. Despite their inherent limitations, their combined use provides a more comprehensive assessment of the antioxidant potential of samples, as these assays are based on different mechanisms of antioxidant action [19].

Chia seeds are rich in phenolic compounds known for their antioxidant properties. These compounds include rosmarinic acid, protocatechuic acid, myricetin, kaempferol, quercetin, caffeic acid, genistein, daidzein, ferulic acid, gallic acid, chlorogenic acid, citric acid, and quinic acid. Rosmarinic acid (RA) stands out as the major phenolic compound in chia seeds [20]. Table 2 lists the phenolic compounds identified in the digested and undigested chia seeds and sprouts from this study. Although the five most abundant phenolic compounds in chia seeds were investigated only rosmarinic acid, rosmarinic acid 3-O-glycoside and caffeic acid were detected in the undigested seed and sprout samples. RA was the main phenolic compound quantified in chia seeds, which is consistent with previous data in the literature [2, 16, 20]. This acid has remarkable biological effects, including antiviral, antibacterial, anticancer, antioxidant, anti-aging, antidiabetic, cardioprotective, hepatoprotective, nephroprotective, antidepressant, antiallergic, and anti-inflammatory activities [21]. In rats with colon cancer, a dose of 5 mg/kg body weight of RA was able to inhibit both tumor formation and growth, in addition to reducing the byproducts of lipid peroxidation and the expression of pro-apoptotic proteins [22]. Furthermore, RA demonstrated antioxidant capacity by scavenging free radicals in hepatic stellate cells (HSCs), stimulating glutathione (GSH) synthesis, and contributing to the inhibition of NF-κB-dependent MMP-2 activity [23]. Synergistically, caffeic acid—although found in a lower initial concentration in the present study—acts as a potent reducing agent and free radical scavenger, contributing to the seed’s antioxidant potential [24].

**Table 2:** Identification of phenolic compounds in undigested and digested chia seeds and sprouts (dry weight - dw)

Phenolic compound (mg/g)	Undigested		Digested			
	GC	GCS	WC	GC	WCS	GCS
Rosmarinic acid	1.08 ± 0.004^aA^	0.41 ± 0.006^b^	ND	0.11 ± 0.002^B^ (10.18%)	ND	< LOD
Rosmarinic acid 3-O-glycoside	1.78 ± 0.005^aA^	0.27 ± 0.002^b^	ND	0.38 ± 0.003^B^ (21.34%)	ND	ND
Caffeic Acid	0.04 ± 0.001^bA^	0.06 ± 0.001^a^	ND	0.37 ± 0.015^B^ (925%)	ND	ND

*WC* whole chia, *WCS* whole chia sprout, *GC* ground chia, *GCS* ground chia sprout, *LOD* limit of detection. The values are expressed as mean ± SD. Undigested samples: different lowercase letters in the same row indicate statistical differences (*p* < 0.05), according to the unpaired Student’s *t*-test. Digested samples: different uppercase letters in the same line indicate a statistical difference (*p* < 0.05) between the digested sample and the respective undigested sample, according to the unpaired Student’s t-test. The bioaccessibility of the phenolic compounds is shown in parentheses

After in vitro gastrointestinal digestion, the levels of RA and rosmarinic acid 3-O-glycoside decreased significantly in the ground seeds (0.11 and 0.38 mg/g/dw, respectively), representing bioaccessibility of 10.18 and 21.34%, respectively. In contrast, these acids were not detected after digestion of the whole samples and GCS. However, caffeic acid exhibited the opposite behavior. While the content in the undigested seeds was low (0.04 mg/g/dw), after the digestion of the ground seeds, there was a significant increase, reaching 0.37 mg/g/dw, representing a bioaccessibility of 925%. Molares et al [25], reported similar behavior in a review conducted to evaluate the application of the INFOGEST 2.0 protocol to food polyphenols. Different studies have reported bioaccessibility values of total and specific phenolic compounds above 100%. Thumann et al [26] also observed higher bioaccessibility of caffeic acid (158%) and lower bioaccessibility of rosmarinic acid (56%) after simulated gastrointestinal digestion of the herbal extract combination. The authors reported that a possible explanation for the reduced levels of rosmarinic acid could be its conversion to caffeic acid through ester cleavage. In the present study, a reduction in RA and an increase in caffeic acid were observed, which could support this association.

The results post-gastrointestinal digestion (Table 2) reinforces the importance of grinding chia seeds for the release of phenolic compounds, as the compounds were only detected in the ground seeds, indicating that the physical structure of the intact seed possibly acts as a barrier, preventing the action of digestive enzymes on the food.

### Fatty Acid Profile

The lipids present in chia seeds are mainly composed of polyunsaturated fatty acids, such as alpha-linolenic acid and linoleic acid [27]. Table 3 presents the profile of the main fatty acids found in chia seeds and sprouts, digested and undigested. Before digestion, in chia seeds and sprouts, α-linolenic acid (C18:3) was predominant, followed by linoleic acid (C18:2), palmitic acid (C16:0), oleic acid (C18:1), and stearic acid (C18:0). A similar lipid profile was found in other studies with chia seeds [2, 28]. Despite the significant reduction in α-linolenic acid during germination, chia sprouts remain an excellent source of omega-3 fatty acids. A 10 g serving of chia seeds or chia sprouts provides approximately 2.60 g and 2.05 g of α-linolenic acid, respectively, reaching the adequate intake (AI) established for adults (1.6 g/day for men and 1.1 g/day for women) [29]. The reduction of fatty acid levels, such as α-linolenic acid, in chia sprouts has also been described in other studies [2, 30]. This decrease during germination may have occurred due to the use of lipids stored in chia seeds as energy sources for the development of the sprout, which can alter the lipid profile [31].

**Table 3:** Main fatty acids found in undigested and digested chia seeds and sprouts (dry weight - dw)

Fatty Acids (g/100 g)	Undigested		Digested			
	GC	GCS	WC	GC	WCS	GCS
Palmitic acid (C16:0)	2.91 ± 0.05^aA^	2.51 ± 0.00^bC^	0.03 ± 0.00^dB^ (1.03%)	0.60 ± 0.03^cB^ (20.61%)	0.04 ± 0.00^dD^ (1.59%)	0.58 ± 0.01^cD^ (23.10%)
Stearic acid (C18:0)	1.21 ± 0.00^aA^	1.07 ± 0.00^bC^	0.01 ± 0.00^dB^ (0.82%)	0.31 ± 0.01^cB^ (25.61%)	0.004 ± 0.00^dD^ (0.37%)	0.28 ± 0.01^cD^ (26.16%)
Oleic acid (C18:1)	2.09 ± 0.15^aA^	1.77 ± 0.01^bC^	0.03 ± 0.00^dB^ (1.43%)	0.43 ± 0.00^cB^ (20.57%)	0.05 ± 0.00^dD^ (2.82%)	0.33 ± 0.00^cD^ (18.64%)
Linoleic acid - LA (C18:2)	7.51 ± 0.10^aA^	6.02 ± 0.01^bC^	0.02 ± 0.00^dB^ (0.26%)	1.09 ± 0.11^cB^ (14.51%)	0.01 ± 0.00^dD^ (0.16%)	0.97 ± 0.04^cD^ (16.11%)
Alpha-linolenic acid - ALA (C18:3)	25.79 ± 0.24^aA^	20.58 ± 0.03^bC^	0.07 ± 0.00^eB^ (0.27%)	3.87 ± 0.04^cB^ (15.00%)	0.07 ± 0.00^eD^ (0.34%)	3.19 ± 0.08^dD^ (15.50%)
Unsaturated fatty acids	35.39 ± 0.02^aA^	28.37 ± 0.00^bC^	0.13 ± 0.00^eB^ (0.36%)	5.40 ± 0.04^cB^ (15.25%)	0.13 ± 0.00^eD^ (0.45%)	4.49 ± 0.04^dD^ (15.82%)
Monounsaturated fatty acids	2.09 ± 0.15^aA^	1.77 ± 0.01^bC^	0.03 ± 0.00^dB^ (1.43%)	0.43 ± 0.00^cB^ (20.57%)	0.05 ± 0.00^dD^ (2.82%)	0.33 ± 0.00^cD^ (18.64%)
Polyunsaturated fatty acids	33.30 ± 0.13^aA^	26.60 ± 0.02^bC^	0.10 ± 0.00^eB^ (0.30%)	4.97 ± 0.06^cB^ (14.92%)	0.08 ± 0.00^eB^ (0.30%)	4.16 ± 0.04^dD^ (15.63%)
Saturated fatty acids	4.12 ± 0.06^aA^	3.59 ± 0.00^bC^	0.05 ± 0.00^dB^ (1.21%)	0.92 ± 0.05^cB^ (22.33%)	0.05 ± 0.00^dD^ (1.39%)	0.86 ± 0.03^cD^ (23.95%)
Total Lipids	39.6 ± 0.08^aA^	32 ± 0.07^bC^	0.19 ± 0.03^eB^ (0.47%)	6.33 ± 0.00^cB^ (15.98%)	0.19 ± 0.00^eD^ (0.59%)	5.36 ± 0.00^dD^ (16.75%)
n-6:n-3	0.29	0.29	0.28	0.28	0.14	0.30

*WC* whole chia, *WCS* whole chia sprout, *GC* ground chia, *GCS* ground chia sprout. The values are expressed as mean ± SD. Undigested samples: different lowercase letters (a, b) in the same row indicate statistical differences (*p* < 0.05), according to the unpaired Student’s *t*-test. Digested samples: different lowercase letters (c, d, e) in the same row indicate statistical differences (*p* < 0.05) according to the one-way non-parametric ANOVA and the Tukey post-hoc test. Uppercase letters in the same row indicate a statistical difference (*p* < 0.05) between the digested sample and the respective undigested sample, according to the unpaired Student’s* t*-test. The bioaccessibility of fatty acids are shown in parentheses

After gastrointestinal digestion, it is observed that grinding is also crucial for the release and bioaccessibility of fatty acids from chia seeds and sprouts. In the digested intact seeds and sprouts, the fatty acids were very low, presenting values of 0.07 g/100 g/dw of ALA for both samples. In contrast, in the crushed samples, the release was higher, reaching 3.87 g/100 g/dw in the seeds and 3.19 g/100 g/dw in the sprouts. A similar result was found by Labanca et al [7], whose samples of intact chia seeds did not show detectable levels of omega-3 or other fatty acids after digestion, indicating that the physical structure of the intact seed possibly hinders enzymatic action. In the present study, all fatty acids showed higher bioaccessibility values in the crushed samples compared to the intact ones. The bioaccessibility, for example, of alpha-linolenic acid in whole seeds was only 0.27%, rising to 15.00% in crushed seeds. A similar behavior was observed in the sprouts, which varied from 0.34% in the whole form to 15.50% in the crushed form. As already mentioned, the low bioaccessibility in whole chia seeds and sprouts can be explained by the rigid physical structure of the chia seed coat, which acts as a mechanical barrier. This blockage prevents gastrointestinal fluids and digestive enzymes from accessing the stored lipid content, hindering its hydrolysis [7]. Although germination reduced the total lipid and alpha-linolenic acid content compared to the seeds, the crushed sprouts had slightly higher bioaccessibility indices than the crushed seeds for most individual fatty acids.

The essential fatty acids LA and ALA are not synthesized by the body and must be consumed through the diet [32]; chia stands out as one of the main plant sources of both nutrients [27]. It has been proposed that the ideal intake ratio of n-6:n-3 should not exceed 10:1 to maintain a healthy balance [33]. However, Western diets are characterized by high n-6 consumption, which raises the n-6:n-3 ratio to a range of 10:1 to 20:1, increasing the risk of developing inflammatory diseases [34]. Chia provides a favorable n-6:n-3 ratio, which can range from 1:3 to 3:1 [2, 35, 36]. Similar results were found in the present study, before and after digestion, for chia seeds and sprouts (Table 3). Thus, the regular consumption of these foods can contribute to the balance between omega-6 and omega-3 fatty acids in the body.

## Conclusions

Based on the results presented, it is concluded that mechanical grinding proved to be a determining factor for the bioaccessibility of phenolic compounds and fatty acids from chia, both in seed and sprout. The physical structure of the whole seed acts as a barrier against the action of digestive enzymes, hindering the release of phenolic compounds and fatty acids. However, germination was not able to increase the phenolic compound content of chia seeds. The gastrointestinal digestion process resulted in a significant increase in caffeic acid due to the degradation of more complex molecules, such as rosmarinic acid, which is the main phenolic compound found in chia. Moreover, the antioxidant capacity significantly increased after the digestion of all samples, especially in the crushed ones. Regarding germination, although the process reduced the initial content of alpha-linolenic acid, chia sprouts were an excellent source of omega-3, exceeding the daily intake recommendations even after this reduction. In summary, grinding allows for greater release of phenolic compounds and fatty acids from chia seeds and sprouts, making it important that these foods are consumed in this manner. However, additional research, especially clinical studies, is still needed to better understand the bioavailability of bioactive compounds of chia seeds and sprouts, both whole and crushed.

## Supplementary Information

Below is the link to the electronic supplementary material.

Supplementary Material 1 (DOCX 358 KB)

## Data Availability

Data will be made available on request.

## References

[1] Hernández-Pérez T, Paredes-López O (2026) Ancient Latin-American food crops: An overview of their nutraceutical and antiobesity peptides. Food Sci Technol Int 32(4):454–464. 10.1177/10820132251319934 PMC13144648 39981629

[2] Salgado VDSCN, Zago L, Fonseca END et al (2023) Chemical composition, fatty acid profile, phenolic compounds, and antioxidant activity of raw and germinated chia (Salvia hispanica L.) seeds. Plant Foods Hum Nutr 78:735–741. 10.1007/s11130-023-01115-0 37856036

[3] Hernández-Pérez T, Valverde ME, Orona-Tamayo D et al (2020) Chia (Salvia hispanica): Nutraceutical Properties and Therapeutic Applications. Proceedings 53(1):17. 10.3390/proceedings2020053017

[4] TaghipourSheshdeh F, Behzadi M, Bashiri S et al (2025) The Effect of Chia Seed on Blood Pressure, Body Composition, and Glycemic Control: A GRADE-Assessed Systematic Review and Dose-Response Meta-Analysis of Randomized Controlled Trials. Nutr Rev 83(3):448–465. 10.1093/nutrit/nuae113 39225983

[5] Han J, Zhang Q, Luo W et al (2022) *In vitro* digestion of whole chia seeds (*Salvia hispanica* L.): Nutrient bioaccessibility, structural and functional changes. Int J Food Sci Technol 57:3727–3734. 10.1111/ijfs.15698

[6] Calvo-Lerma J, Paz-Yépez C, Asensio-Grau A et al (2020) Impact of processing and intestinal conditions on in vitro digestion of chia (Salvia hispanica) seeds and derivatives. Foods 9:290. 10.3390/foods9030290 PMC7143566 32150813

[7] Labanca AR, Svelander C, Alminger M (2019) Effect of particle size of chia seeds on bioaccessibility of phenolic compounds during in vitro digestion. Cogent Food Agric 5:1694775. 10.1080/23311932.2019.1694775

[8] Baldelli A, Aguilera JM (2025) Size reduction and particle size influence the quantification of phenolic-type compounds and antioxidant activity in plant food matrices. Trends Food Sci Technol 162(5):1–7

[9] Lemmens E, Moroni AV, Pagand J et al (2019) Impact of Cereal Seed Sprouting on Its Nutritional and Technological Properties: A Critical Review. Compr Rev Food Sci Food Saf 18(1):305–328. 10.1111/1541-4337.12414 33337026

[10] Samtiya M, Aluko RE, Dhewa T et al (2021) Potential health benefits of plant food-derived bioactive components: an overview. Foods 10:839. 10.3390/foods10040839 PMC8068854 33921351

[11] Abdel-Aty AM, Elsayed AM, Salah HA et al (2021) Egyptian chia seeds (*Salvia hispanica* L.) during germination: upgrading of phenolic profile, antioxidant, antibacterial properties and relevant enzymes activities. Food Sci Biotechnol 30:723–734. 10.1007/s10068-021-00902-2 PMC8144251 34123468

[12] Pająk P, Socha R, Broniek J et al (2019) Antioxidant properties, phenolic and mineral composition of germinated chia, golden flax, evening primrose, phacelia and fenugreek. Food Chem 275:69–76. 10.1016/j.foodchem.2018.09.081 30724250

[13] Menezes MCS, Miyahira RF, Inada KOP et al (2025) Adverse influence of chia seed germination on iron bioaccessibility and uptake by Caco-2 cells. 71:107244. 10.1016/j.fbio.2025.107244

[14] Salgado VDSCN, Zago L, Antunes AEC et al (2022) Chia (Salvia hispanica L.) seed germination: a brief review. Plant Foods Hum Nutr 77:485–494. 10.1007/s11130-022-01011-z 36083408

[15] Bashmil YM, Dunshea FR, Appels R et al (2024) Bioaccessibility of phenolic compounds, resistant starch, and dietary fibers from Australian green banana during in vitro digestion and colonic fermentation. Molecules 29:1535. 10.3390/molecules29071535 PMC11013930 38611814

[16] Pellegrini M, Lucas-Gonzalez R, Sayas-Barberá E et al (2018) Bioaccessibility of phenolic compounds and antioxidant capacity of chia (*Salvia hispanica* L.) seeds. Plant Foods Hum Nutr 73:47–53. 10.1007/s11130-017-0649-7 29188413

[17] Hernández-Olivas E, Muñoz-Pina S, Andrés A et al (2023) The impact of age-related digestive disorders on in vitro digestibility of macronutrients and bioaccessibility of minor components of chia seeds. Food Res Int 169:112874. 10.1016/j.foodres.2023.112874 37254324

[18] Serena-Romero G, Ignot-Gutiérrez A, Conde-Rivas O et al (2023) Impact of In Vitro Digestion on the Digestibility, Amino Acid Release, and Antioxidant Activity of Amaranth (*Amaranthus cruentus* L.) and Cañihua (*Chenopodium pallidicaule* Aellen) Proteins in Caco-2 and HepG2 Cells. Antioxid (Basel) 12(12):2075. 10.3390/antiox12122075 PMC10740650 38136195

[19] Schaich M, Tian X, Xie J (2015) Hurdles and pitfalls in measuring antioxidant efficacy: A critical evaluation of ABTS, DPPH, and ORAC assays. J Funct Foods 14:111–125. 10.1016/j.jff.2015.01.043

[20] Alcântara MA, de Lima Brito Polari I, de Albuquerque Meireles BRL et al (2019) Effect of the solvent composition on the profile of phenolic compounds extracted from chia seeds. Food Chem 275:489–496. 10.1016/j.foodchem.2018.09.133 30724224

[21] Nadeem M, Imran M, Aslam Gondal T et al (2019) Therapeutic Potential of Rosmarinic Acid: A Comprehensive Review. Appl Sci 9(15):3139. 10.3390/app9153139

[22] Venkatachalam K, Gunasekaran S, Namasivayam N (2016) Biochemical and molecular mechanisms underlying the chemopreventive efficacy of rosmarinic acid in a rat colon cancer. Eur J Pharmacol 791:37–50. 10.1016/j.ejphar.2016.07.051 27565219

[23] Lu C, Zou Y, Liu Y et al (2017) Rosmarinic acid counteracts activation of hepatic stellate cells via inhibiting the ROS-dependent MMP-2 activity: involvement of Nrf2 antioxidant system. Toxicol Appl Pharmacol 318:69–78. 10.1016/j.taap.2017.01.008 28115189

[24] Pavlíková N (2022) Caffeic acid and diseases-mechanisms of action. Int J Mol Sci 24:588. 10.3390/ijms24010588 PMC9820408 36614030

[25] Morales D, Iriondo-DeHond A, Fernández-Tomé S (2025) Application of the INFOGEST 2.0 standardized method to study the behavior of phenolic compounds throughout gastrointestinal digestion. Food Chem 492(Pt 2):145531. 10.1016/j.foodchem.2025.145531 40675074

[26] Thumann TA, Pferschy-Wenzig EM, Aziz-Kalbhenn H, Ammar RM, Rabini S, Moissl-Eichinger C, Bauer R (2020) Application of an in vitro digestion model to study the metabolic profile changes of an herbal extract combination by UHPLC–HRMS. Phytomedicine 71 10.1016/j.phymed.2020.153221 32447246

[27] Knez Hrnčič M, Ivanovski M, Cör D, Knez Ž (2020) Chia seeds (*Salvia hispanica* L.): an overview-phytochemical profile, isolation methods, and application. Molecules 25:11. 10.3390/molecules25010011 PMC6994964 31861466

[28] Shahjadee UF, Hossain MH, Ethane NJ et al (2026) Impact of germination on the nutritional composition and bioactive properties of Bangladeshi Salvia hispanica L. seeds. Food Humanity 101209. 10.1016/j.foohum.2026.101209

[29] National Institutes of Health (NIH) - Office of Dietary Supplements Omega-3 Fatty Acids: Fact Sheet for Health Professionals. https://ods.od.nih.gov/factsheets/Omega3FattyAcids-HealthProfessional. Accessed 26 June 2026

[30] Ghafoor K, Juhaimi AF, Özcan MM et al (2022) The effect of boiling, germination and roasting on bioactive properties, phenolic compounds, fatty acids and minerals of chia seed (salvia hispanica l.) and oils. Int J Gastronomy Food Sci 27:100447. 10.1016/j.ijgfs.2021.10044

[31] Coelho MS, Salas-Mellado MDM (2015) Effects of substituting chia (Salvia hispanica L.) flour or seeds for wheat flour on the quality of the bread. LWT - Food Sci Technol 60:729–736. 10.1016/j.lwt.2014.10.033

[32] Kapoor B, Kapoor D, Gautam S et al (2021) Dietary polyunsaturated fatty acids (PUFAs): uses and potential health benefits. Curr Nutr Rep 10:232–242. 10.1007/s13668-021-00363-3 34255301

[33] Institute of Medicine (2005) Dietary reference intakes for energy, carbohydrate, fiber, fat, fatty acids, cholesterol, protein, and amino acids. National Academy, Washington (DC) 10.1016/s0002-8223(02)90346-9 12449285

[34] Bishehkolaei M, Pathak Y (2024) Influence of omega n-6/n-3 ratio on cardiovascular disease and nutritional interventions. Hum Nutr Metab 200275. 10.1016/j.hnm.2024.200275

[35] Farag MA, Khattab AR, Farghal HH et al (2025) Gas chromatography/mass spectrometry-based metabolite profiling of chia and quinoa seeds in comparison with wheat and oat. Phytochem Anal 36(8):2301–2320. 10.1002/pca.3398 38802070

[36] Pandurangan SB, Al-Maiman SA, Al-Harbi LN et al (2020) Beneficial fatty acid ratio of Salvia hispanica L. (Chia Seed) potentially inhibits adipocyte hypertrophy and decreases adipokines expression and inflammation in macrophage. Foods 9:368. 10.3390/foods9030368 PMC7143507 32235695

