# Supplementary material for: Bioaccessibility of Phenolic Compounds and Polyunsaturated Fatty Acids in Whole and Ground Chia Seeds and Sprouts
Source: Plant Foods Hum Nutr. 2026 Oct 14;81(4):129. doi: 10.1007/s11130-026-01577-y (PMC13668980; doi:10.1007/s11130-026-01577-y)
Supplement: Supplementary file 1 [file 11130_2026_1577_MOESM1_ESM.docx]

**Materials and Methods**

**Experimental Design**

Analyses of total phenolic compounds (Folin- Ciocalteu), identification of phenolic compounds (HPLC), antioxidant capacity (ABTS, FRAP, and DPPH), and fatty acid profile were performed on samples of whole chia (WC), whole chia sprout (WCS), ground chia (GC), and ground chia sprout (GCS), as shown in the experimental design of the study (Fig. 1). Figure 1: Experimental design of the study

**Sample Preparation**

The chia seeds (WC) were purchased from a local store in the municipality of Rio de Janeiro. The germination process was carried out according to Nadtochii et al. [1], with modifications. Initially, the seeds were soaked in filtered water for 10 minutes to promote complete hydration of the mucilage. Next, the water was drained with the help of a plastic sieve. Subsequently, the seeds were distributed in plastic trays lined with filter paper. Germination occurred at room temperature (20–25 °C) and relative humidity of approximately 70%, under a lighting scheme of 12 hours of light (LED) and 12 hours of darkness. The humidity was maintained by spraying filtered water twice a day (morning and evening). The germination process lasted for 72 hours (3 days) (WCS). The chia seeds were crushed using a blender with a power of 450 watts until their content was homogeneous and without the presence of whole seeds (GC). The chia sprouts were dried in an oven with air circulation and renewal (Tecnal) at 50º C for 8 hours and ground in a blender with a power of 450 watts [2] This sample (GCS) was the only one subjected to oven drying. All samples were stored frozen (-20º C) until the analyzes were conducted.

**Simulated gastrointestinal digestion**

The *in vitro* gastrointestinal digestion of the WC, GC, WCS, and GCS was carried out based on the INFOGEST 2.0 protocol, with modifications [3]. Initially, the activities of the digestive enzymes (pepsin and pancreatin) and the concentrations of bile salts were determined through standardized tests. Stock solutions of electrolytes for simulated salivary (SSF), gastric (SGF), and intestinal (SIF) fluids were also prepared at a concentration of 1.25x. Gastrointestinal digestion was carried out in three phases: oral, gastric, and intestinal. In the oral phase, 5g of each sample were diluted in SSF, human amylase, and calcium chloride (CaCl_2_) in a 1:1 (w/w) ratio, manually macerated to disrupt the tissue structure to obtain a swallowable paste, and incubated at 37ºC in a water bath with agitation for 2 minutes. The resulting oral bolus was diluted in SGF and pepsin in a 1:1 (v/v) ratio and incubated under agitation in a water bath at 37ºC (pH 3) for 2 hours. The gastric chyme was then diluted in a 1:1 (v/v) ratio with SIF, bile salts, and pancreatin and incubated again in a water bath at 37ºC (pH 7) for 2 hours. After digestion, the samples were centrifuged at 21.200 x g, at 4 °C, for 30 minutes to obtain the bioaccessible fraction [4].

**Bioaccessibility Index**
The effect of *in vitro* gastrointestinal digestion on the phenolic compounds and fatty acids of the samples was evaluated using the bioaccessibility index (BI), according to the following equation:

BI = CD/IC x 100
CD = Concentration after digestion
IC = Initial concentration

**Extraction of phenolic compounds from undigested samples**
The extraction of phenolic compounds was carried out according to the protocol described by Abdel-Aty et al. [5], with modifications. One gram of chia (GC and GCS) was suspended in 10 mL of a methanol/water solution (80:20, v/v). The samples were kept under agitation for 24 hours at room temperature (20°C – 25°C). Subsequently, the material was centrifuged at 21.200x g at 4 °C for 30 minutes. In the end, the supernatant was collected for the determination of total phenolic content and antioxidant capacity. The results were expressed on a dry base.

**Total Phenolic Compounds (TPC) Content**

The determination of TPC for undigested (GC and GCS) and digested (WC, GC, WCS, and GCS) samples was performed according to the Folin-Ciocalteu method described by Singleton et al. [6], with adaptation for microvolumes. In a 96-well microplate, 20 μL of the sample or standard and 100 μL of the 10% Folin-Ciocalteu reagent were added. After 5 minutes, 75 μL of 7.5% sodium carbonate were added. The contents of the microplate were homogenized and kept in the dark for 40 minutes. The absorbance reading was performed at 740 nm, using a microplate reader -Victor X3 (PerkinElmer, Massachusetts, USA). The results were expressed in milligrams of gallic acid equivalents per gram (mg GAE/g) of dry basis.

**Identification of Phenolic Compounds**

The identification of phenolic compounds in undigested (GC and GCS) and digested (WC, GC, WCS, and GCS) samples was carried out using a high-performance liquid chromatography (HPLC) system (Shimadzu®, Japan), which included two parallel LC-40D XR pumps, an automatic injector SIL-40C XR, a CTO-40C column oven, a CBM-40 system controller, a DGU-405 degasser, and a SPD-M40 diode array detector (DAD). The chromatographic separation of phenolic compounds was performed using a Shim-pack GIST C18 reverse phase column (5 μm, 250 mm × 4.6 mm, Shimadzu®) with a Shim-pack GIST C18 guard column (5 μm, 10 mm × 4.0 mm, Shimadzu®). The mobile phase consisted of a gradient of 1% formic acid in water (eluent A) and acetonitrile (eluent B), at a flow rate of 1.0 mL/min, following a gradient profile of 0−20 min, 5−30% B; 20−30 min, 30−55% B; 30−38 min, 55−90% B; 38−40 min, 90% B; and then returning to initial conditions. The compounds were monitored by DAD from 190 to 600 nm. The temperature was maintained at 40°C, and the injection volume was 10 μL. Before injection, the samples were filtered using a 0.45 μm PVDF filter (Filtrilo®, Colombo, Brazil). The identification was performed by comparison with the retention time and the absorption spectrum of the standards (3-*O*-caffeoylquinic, 5-*O*-caffeoylquinic, caffeic, ferulic, sinapic, rosmarinic, 3,4-dihydroxycinnamic, dihydrocaffeic, p-coumaric, dihydroferulic, trans-cinnamic, 2-hydroxycinnamic, and dihydro-p-coumaric acids) [5]. The quantification was performed using calibration curves of the respective standards. Rosmarinic acid glycoside was identified based on its chromatographic and spectrometric characteristics and quantified using the calibration curve of rosmarinic acid, as an authentic standard for the glycosylated derivative was not available. Therefore, its concentration was expressed as rosmarinic acid equivalents. The data were acquired using Lab Solutions software (Shimadzu Corporation®, version 5.111, 2008–2021).

The limits of detection (LOD) and quantification (LOQ) were estimated from the calibration curves using the residual standard deviation of the regression and the slope, according to LOD = 3.3σ/S and LOQ = 10σ/S, respectively (Table S1). Calibration curves were prepared using caffeic acid and rosmarinic acid standards at a concentration of 30 µg/mL, using injection volumes of 1, 2.5, 5, 10, and 20 µL, corresponding to calibration levels of 0.03, 0.075, 0.15, 0.30, and 0.60 µg/injection. LOD and LOQ values were subsequently expressed as mg/g of dry weight on the sample preparation procedure (1 g of sample extracted to 10 mL) and a 10-µL injection volume, without further dilution of the extracts.

**Table S1** Calibration curve parameters, limits of detection (LOD) and limits of quantification (LOQ) for the phenolic standards used in quantification by HPLC-DAD.

| **Compound** | **Calibration range (µg/injection)** | **Equation** | **R²** | **LOD (mg/g)** | **LOQ (mg/g)** |
| --- | --- | --- | --- | --- | --- |
| Caffeic acid | 0.03–0.60 | y = 1,805,286.88x + 2,726.33 | 0.9999 | 0.00844 | 0.02558 |
| Rosmarinic acid | 0.03–0.60 | y = 3,203,203.85x − 12,704.09 | 0.9994 | 0.02105 | 0.06380 |

*LOD and LOQ values expressed as mg/g dry sample were calculated based on the sample preparation procedure (1 g of sample extracted to 10 mL) and a 10-µL injection volume, without further dilution of the extracts.

**Antioxidant Capacity**

The antioxidant capacity of the undigested (GC and GCS) and digested (WC, GC, WCS, and GCS) samples were determined using three different methods, each based on distinct mechanisms of action. All assays were performed in 96-well microplates, and the absorbance was measured by spectrophotometry using a microplate reader (PerkinElmer, Massachusetts, USA). The antioxidant capacity was determined by the ferric reducing antioxidant power (FRAP) method, as described by Müller et al. [7], with readings at 595 nm, and the results were expressed in ferrous sulfate equivalents (μM). The antioxidant capacity equivalent to the ABTS+ radical (ABTS) and the effect of eliminating the free radical 1,1-diphenyl-2-picrylhydrazyl (DPPH) were determined according to Al-Duais et al. [8], with readings at 730 nm for ABTS and 517 nm for DPPH.

**Lipid Extraction and Fatty Acid Profile**

The lipids from the undigested samples (GC and GCS) were extracted according to the Bligh & Dyer method [9], with modifications. About 1 g of each sample was subjected to extraction using chloroform (5mL), methanol (10mL), and water (4mL). The tubes were placed in a shaker for 30 minutes. Next, 5mL of chloroform and 5mL of 1.5% sodium sulfate solution were added. The lower phase of the mixture was subjected to evaporation. After the evaporation of the solvent, the extracted fat content was weighed. For the digested samples (WC, GC, WCS, and GCS), the methodology described by Tormási and Abrankó [10] was used. In summary, 5 mL aliquots of intestinal fluid were homogenized with 6.25 mL of chloroform. The chloroform phase was transferred to a round-bottom flask and, after the solvent was evaporated, the dry matter content was determined by drying in an oven at 103 ± 1 °C. After extraction, the lipid fraction was subjected to a methylation reaction, and the obtained fatty acid methyl esters (FAMEs) were analyzed by gas chromatography -Thermo Fisher- coupled with a Flame Ionization Detector (FID). A TR-FAME capillary column (120 m in length × 0.25 mm in internal diameter, film thickness of 0.25 μm, part number: 260M166L Thermo Fisher) was used for the gas chromatography system. The oven temperature was set as follows: from 100 °C to 240 °C at 3 °C/min. The injector temperature was 225ºC in split mode. Helium was used as the carrier gas at a linear flow rate. The FAMEs were quantitatively measured using the internal standard C11:0. The total fat was calculated as the sum of all fatty acids. Saturated and monounsaturated fats were calculated as the sum of their respective fatty acids. The results of this analysis were expressed in grams of fatty acids per 100g of total fat found individually in each sample (g/100g).

**Statistical Analysis**

The analyzes were performed in triplicate for all determinations. The results were presented as mean and standard deviation. The data were statistically analyzed using analysis of variance (ANOVA) and unpaired Student's t-test, employing Tukey's test to verify significant differences. The significance level adopted was 5% (p < 0.05), using the statistical software GraphPad Prism 9.

**References**

1. Nadtochii LA, [Kuznetcova DV](https://dspace.emu.ee/browse/author?startsWith=Kuznetcova,%20D.V.), [Proskura AV](https://dspace.emu.ee/browse/author?startsWith=Proskura,%20A.V.) et al (2019) Investigation of various factors in sprout germination of chia (Salvia hispanica L.) seeds. Agric Res 17:1390–1400. <https://doi.org/10.15159/ar.19.128>
2. Gómez-Favela MA, Gutiérrez-Dorado R, Cuevas-Rodríguez EO et al (2017) Improvement of chia seeds with antioxidant activity, GABA, essential amino acids, and dietary fiber by controlled germination bioprocess. Plant Foods Hum Nutr 72:345–352. https://doi.org/10.1007/s11130-017-0631-4
3. Brodkorb A, Egger L, Alminger M et al (2019) INFOGEST static in vitro simulation of gastrointestinal food digestion. Nat Protoc 14:991–1014. <https://doi.org/10.1038/s41596-018-0119-1>
4. Corte-Real J, Richling E, Hoffmann L, Bohn T (2014) Selective factors governing in vitro β-carotene bioaccessibility: negative influence of low filtration cutoffs and alterations by emulsifiers and food matrices. Nutr Res 34(12):1101-10. https://doi.org/10.1016/j.nutres.2014.04.010
5. Abdel-Aty AM, Elsayed AM, Salah HA et al (2021) Egyptian chia seeds (Salvia hispanica L.) during germination: upgrading of phenolic profile, antioxidant, antibacterial properties and relevant enzymes activities. Food Sci Biotechnol 30:723–34. https://doi.org/10.1007/s10068-021-00902-2
6. Singleton VL, Orthofer R, Lamuela-Raventós RM (1999) Analysis of total phenols and other oxidation substrates and antioxidants by means of Folin-Ciocalteu reagent. Methods Enzymol 299:152–178. https://doi.org/10.1016/S0076-6879(99)99017-1
7. Müller L, Gnoyke S, Popken AM et al (2010) Antioxidant capacity and related parameters of different fruit formulations. LWT - Food Sci Technol 43:992–999. https://doi.org/10.1016/J.LWT.2010.02.004
8. Al-Duais M, Müller L, Böhm V et al (2009) Antioxidant capacity and total phenolics of Cyphostemma digitatum before and after processing: use of different assays. Eur Food Res Technol 228:813–821. <https://doi.org/10.1007/s00217-008-0994-8>
9. Bligh EG, Dyer WJ (1959) A rapid method of total lipid extraction and purification. Can J Biochem Physiol 37:911–917. https://doi.org/10.1139/o59-099
10. Tormási J, Abrankó L (2021) Assessment of fatty acid-specific lipolysis by in vitro digestion and GC-FID. Nutrients 13:3889. <https://doi.org/10.3390/nu13113889>
